# Outcomes of a Decision-Making Capacity Assessment Model at the Grey Nuns Community Hospital

**DOI:** 10.3390/ijerph19031560

**Published:** 2022-01-29

**Authors:** Lesley Charles, Utkarsha Kothavade, Suzette Brémault-Phillips, Karenn Chan, Bonnie Dobbs, Peter George Jaminal Tian, Sharna Polard, Jasneet Parmar

**Affiliations:** 1 Division of Care of the Elderly, Department of Family Medicine, University of Alberta, Edmonton, AB T6G 2T4, Canada; utkarsha@ualberta.ca (U.K.); kchan1@ualberta.ca (K.C.); Bdobbs@ualberta.ca (B.D.); Petergeo@ualberta.ca (P.G.J.T.); Jasneet.Parmar@albertahealthservices.ca (J.P.); 2 Department of Occupational Therapy, University of Alberta, Edmonton, AB T6G 2G4, Canada; Suzette.bremault-phillips@ualberta.ca; 3 Library Services, Covenant Health, Edmonton, AB T6L 5X8, Canada; Sharna.Polard@covenanthealth.ca

**Keywords:** decision-making capacity assessment, care of the elderly, geriatrics

## Abstract

BACKGROUND. With an increasing elderly population, the number of persons with dementia is expected to increase and, consequently, the number of persons needing decision-making capacity assessments (DMCA) is too. However, many healthcare professionals do not feel ready to provide DMCAs. Since 2006, we implemented a DMCA Model that includes a care pathway, worksheets, education, and mentoring. The objective of this study was to assess the impact of the utilization of this patient-centered DMCA model on the need for Capacity Interviews. METHODS. This was a retrospective quality assurance chart review of patients referred for DMCA to the Geriatric Service at the Grey Nuns Community Hospital from 2006–2020. The Geriatric Service is run by Family Physicians with extra training in Care of the Elderly. We extracted patient demographics, elements of the DMCA process, and whether Capacity Interviews were performed. We used descriptive statistics to summarize the data. RESULTS. Eighty-eight patients were referred for DMCAs, with a mean age of 76 years (SD = 10.5). Dementia affected 43.2% (38/88) of patients. Valid reasons for conducting a DMCA were evident in 93% (80/86) of referrals, and DMCAs were performed in 72.6% (61/84). 85.3% (58/68) of referrals identified the need for DMCA in two to four domains, most commonly accommodation, healthcare, and finances. Two to three disciplines, frequently social workers and occupational therapists, were involved in conducting the DMCAs for 67.2% (39/58) of patients. The Capacity Assessment Process Worksheet was used 63.2% of the time. Capacity Interviews were conducted in only 20.7% of referrals. Following the DMCAs, 48.2% (41/85) of those assessed were deemed to lack capacity. CONCLUSION. This study suggests that the DMCA Model implemented has decreased the need for Capacity Interviews while simultaneously respecting patient autonomy. This is an important finding as DMCAs carried out following this process reduced the need for both a Capacity Interview and declarations of incapacity while simultaneously respecting patient autonomy and supporting patients in their decisions in accordance with the legislation.

## 1. Introduction

Capacity is a person’s ability to make informed decisions. This involves understanding information and making a choice consistent with one’s preferences and beliefs [1,2,3,4]. A lack of capacity can lead to improper decision-making, which can result in harm to self and others. Over the past few years, Canada’s elderly population has been increasing and, with it, the number of patients with cognitive impairments either due to dementia, other neurological disorders, or chronic medical conditions [3,5,6,7,8]. COVID-19 and associated physical illness or stress on mental health can also potentially compromise a person’s ability to make sound decisions. Consequently, health care professionals (HCPs) are required to conduct far more decision-making capacity assessments (DMCAs) than ever before. A DMCA is an assessment of varying degrees of complexity conducted by HCPs to determine whether there is sufficient evidence to declare a person incapable of managing his or her affairs [9].

While numerous assessment instruments are available for determining a patient’s decision-making ability [4], some HCPs are either uncomfortable or unprepared to conduct a capacity assessment [2,10]. A DMCA Model was developed based on clinical best-practices, ethical guidelines, and legislative acts in Alberta to facilitate determination of decision-making capacity [11]. A process was proposed with front-end screening/problem solving, a well-defined standardized assessment, and a definition of team members’ roles. A care map was developed based on this process. Documentation was developed consisting of a capacity assessment database and patient interview for formal capacity assessment. In 2006, this DMCA Model was introduced and implemented at various health care facilities in Alberta, Canada. The DMCA Model consists of three steps: (1) initial assessment of identifying reasons for assessment; (2) in-depth assessment which involves problem-solving using cognitive and functional testing; and (3) a Capacity Interview, which, is conducted when problems cannot be solved by less intrusive means [11]. Workshops aimed at educating participants on the application of the DMCA Model have been provided to allied HCPs and physicians since 2006 by family physicians certified in the Care of the Elderly and HCPs. The DMCA Model and education have been adapted for different disciplines and settings [10,11,12,13]. A feasibility study looking at three acute-care sites in Edmonton confirmed that this process addressed the issues of lack of knowledge, skill set, etc. [11].

There is little literature on the outcome of DMCAs and what is available points to poor documentation being a weakness [3,4]. This study was undertaken to determine the effectiveness of the DMCA Model by looking at adherence to it and related need for a Capacity Interview. The results of a pilot study involving 12 DMCAs [11] demonstrated a decreased need for Capacity Interviews by 80% if the model was used with fidelity. This is on the assumption that prior to the introduction of the DMCA Model all capacity assessments involved Capacity Interview and was the usual practice at the time [11].As the pilot involved only a limited number of assessments, we wished to explore the impact in more detail. This paper provides results from DMCA evaluation outcomes requested and conducted at the Grey Nuns Community Hospital from 2006–2020, a site where extensive education had been provided on the DMCA Model and DMCAs conducted. This study assesses the impact of utilizing a patient-centered process and standardized approach to DMCAs on the need for the Capacity Interview and preservation of patient autonomy.

## 2. Methods

### 2.1. Study Design and Setting

This was a retrospective quality assurance chart review (Health Research Ethics Board Study ID No. Pro00098216). We reviewed the charts of all in-patients referred for DMCAs to the Geriatric Service at the Grey Nuns Community Hospital from 2006–2020. This is a 500-bed community hospital with 150 medicine beds. The Geriatric Service is run by Family Physicians with extra training in Care of the Elderly. Most of the referrals were from inpatient medicine, but referrals were also received from surgery, palliative care, and psychiatry. The 88 patients represented patients from any of the aforementioned services that the attending teams required assistance in DMCA. The Geriatric Service would consult on these cases and offer advice to the Attending Team.

### 2.2. Process of DMCA

**Figure 1:**
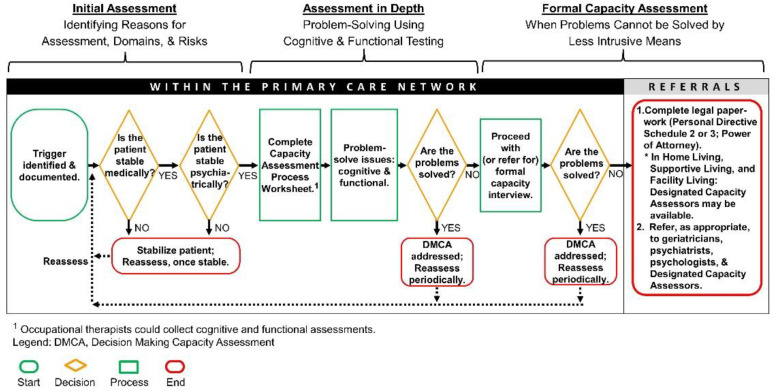
Capacity assessment process.

The initial assessment starts with the evaluation of the validity of the trigger for the DMCA referral. A trigger is an event or circumstance that potentially places a patient, or others, at risk and that seems to be caused by impaired decision-making. Common medical examples are refusal of medical treatment such as chemotherapy or surgery, e.g., amputation. Common personal examples are refusal of referral for higher level of care despite safety issues like wandering, leaving the stove on, not remembering to eat, or eating spoiled food. When the trigger is valid and the patient is medically and psychiatrically stable, a more in-depth assessment follows. The social worker (where available) completes a Capacity Assessment Process Worksheet, including the identification of the domains involved (e.g., accommodation, healthcare). The team then addresses the problems of the patient, making referrals when appropriate. An example would be where the patient living in an apartment is leaving the stove on with risk to them and others. The patient is agreeable to Meals on Wheels and the stove being switched off. It does not mean that the patient is necessarily capable but the problem is solved by less intrusive means. When the problem is not solved, a formal Capacity Interview is conducted, after which problems are further addressed, additional referrals potentially made, and legal paperwork completed as required. See Figure 1 [13].

This is a three-step process. There is some overlap with Comprehensive Geriatric Assessment (CGA), which involves assessing current problems, geriatric syndromes, past medical history and medications, family/social/functional/cognitive history, and cognitive and physical examination. Provided there is suitable trigger for DMCA and the patient is stable, the CGA contributes to data-gathering in the second step, which is needed for problem solving.

### 2.3. Outcome Measures and Analysis

We extracted data on patient demographics: (1) age and sex, (2) living arrangements, (3) diagnosis of dementia; and on the process of DMCA: (1) number of DMCAs requested and performed, (2) validity of the trigger, (3) domains and disciplines involved, (4) referrals made, (5) use of the Capacity Assessment Process Worksheet, (6) team conferences held, (7) Capacity Interviews performed, (8) and final dispositions whether capable or not. We reviewed the charts and extracted data on these variables. Some variables had values in all patients; other variables were not noted or were missing in some patients. Hence, the denominators of the variables varied depending on the available data; sample size ranged from 58 to 88. We used descriptive statistics to summarize the data.

## 3. Results

Eighty-eight patients were referred for DMCAs in 2006–2020. The mean age was 76 years (SD = 10.5; range: 49–98), with 51% females (45/88). Most (97.5%) patients lived at home. Dementia affected 43.2% (38/88) of patients, and 13.6% had an unspecified cognitive impairment (See Table 1). After initial assessments of the 88 referrals, 93.0% (80/86) had valid triggers. Subsequently, 72.6% (61/84) DMCAs were performed.

### 3.1. Domains Identified in the Patients

85.3% (58/68) of the patients were referred to as a result of decision-making concerns in two to four domains. The most common domains identified were accommodation (88.2%, 60/68), healthcare (83.8%, 57/68), and finances (61.8%, 42/68).

### 3.2. Disciplines Involved and Referrals Made

In 58 of the 88 patients referred for DMCA, we had data on disciplines involved. The DMCAs involved two to three disciplines for 67.2% (39/58) of the cases. The disciplines most commonly involved in the DMCAs were social workers (81.0%, 47/58), occupational therapists (53.4%, 31/58), physicians (22.4%, 13/58), and nursing (LPN, RN, NP, 19%, 11/58). A total of 50 referrals were made during the DMCA process in the files reviewed, with one (61.4%, 54/88) or two (27.3%, 24/88) referrals being most common. These referrals were most frequently made to geriatricians (87.5%, 77/88) and social workers (25.0%, 22/88).

### 3.3. Process

Capacity Assessment Process Worksheets were used in 63.2% (55/87) of the referrals, team conferences were used 28.9% (24/83) of the time, and a Capacity Interview was required in 20.7% (18/87) of the cases. Following the DMCA, 48.2% (41/85) of patients were deemed to lack capacity, 23.5% (20/85) were deemed to have capacity, and 8.2% (7/85) were deemed to not require a capacity assessment. See Table 2.

## 4. Discussion

The DMCA Model has been implemented in an acute care clinical setting at the Grey Nuns Community Hospital since 2006. From 2006–2020, 88 referrals for DMCA were received by the geriatric service. DMCAs were carried out in 72.6% of the cases. This compares with other studies where they carried out DMCA requests ranging from 4–206 [2,14,15,16,17,18]. However, our number only represents referrals that were directed to the geriatric service for geriatric or designated capacity assessor (social worker, occupational therapist, RN, or psychiatric nurse trained and designated under the Adult Guardianship and Trusteeship Act to conduct DMCA) [19]) input and does not include DMCAs handled by an attending team or cases referred to psychiatry or for psychology opinion.

As DMCAs can be burdensome and invasive for patients, it is essential that unnecessary DMCAs are reduced. There are few published reports where DMCAs were conducted on more than one occasion on the same patient [14,15] due to referrals being made for multiple incapacities. This was not the case in our study. Our results are significant as use of the DMCA Model reduced the need for multiple DMCAs on a single patient, thereby decreasing the burden and stress on HCPs and patients. The DMCA Model and care map provide a standardized, clear, and effective pathway that minimizes the need for multiple DMCAs [11]. The DMCA Model offers HCPs, inter-professional teams, and organizations a best-practice and implementation approach to DMCAs [20].

Domains in which patients require DMCAs vary. Results from our study identified accommodation (88.2%) as the predominantly referred domain, closely followed by healthcare (83.8%) and financial matters (61.8%), with these results consistent with previously published reports (accommodation 63%, financial 22%, and medical 12%) [21]. With appropriate medico-social intervention and proper utilization of the DMCA Model, patients were protected from further harm through the enactment of personal directives or enduring power of attorney, or application for guardianship/trusteeship.

Dementia, an irreversible neurodegenerative disorder [3,5,14,22], is associated with a progressive decline in decision-making capacity. Due to the nature of this condition, frequent and multiple referrals are sent for DMCAs [14]. However, no significant difference in DMCA referrals was observed in our study regardless of the presence (43.2% had been diagnosed with dementia) or absence of dementia. This could be an under-representation as DMCA often comes to a head with acute decompensation and admission to hospital, where only then is the diagnosis of dementia made.

A multidisciplinary approach to patient care and determination of decision-making ability can be very effective in DMCAs. At the Grey Nuns Community Hospital, where such an approach has been adopted, social workers (81%) were the most common discipline involved in DMCAs. These findings confirm that social workers play a vital role in the (i) initial evaluation of patients’ decision-making ability; (ii) gathering of collateral information by communicating with family, friends, financial institutes, and long -term care facilities; and (iii) communicating findings with the attending team to resolve underlying issues in ways that preserve patient integrity and rights [23] and, if required, referring patients for a Capacity Interview. The literature supports the involvement of a variety of professionals in DMCA, ranging from psychiatrists to geriatric services, to social workers [14,15,16,17,18]. One study [15] had a multidisciplinary team (MDT) consisting of various medical professionals ranging from gastroenterologists to speech and language therapists. This would be most akin to our DMCA Model. In another study [2], DMCA was carried out by pre-doctoral psychology interns who received training in DMCA. Surprisingly, physicians, given their pivotal role in enactments of incapacity, were only involved in 22.4% of DMCAs. However, given that only 20.7% of patients referred for DMCAs required an actual Capacity Interview, this would be appropriate, with the team collecting the information and solving the problem before a Capacity Interview was needed.

Referrals for Capacity Interviews were made frequently to geriatricians. While legally all medical professionals can be involved in DMCAs, many do not have the necessary skills and training needed to perform them and, as a result, often refer patients to specialists [14,24]. This finding is notable as many physicians are often less involved in DMCA due to a lack of experience, training and skills in performing a formal DMCA [2,10,14,16,25]. The need for physicians, such as geriatricians and general practitioners, to perform DMCA is on the rise due to the increasing elderly population and an associated decline in their decision-making capacity. The role of geriatric physicians is crucial because of their frequent interaction with elderly patients, their awareness of elderly patients’ clinical conditions, and their ability to keep up with elderly patients’ progress over time [10,14].

The results of capacity assessments can vary significantly. Following DMCAs conducted using the DMCA Model and care map, 48.2% (41/85) of our patients were deemed to lack capacity. 23.5% had capacity, for 8.2% it was decided not to proceed with DMCA, and 20% were unknown. Comparatively, the proportion of patients who were deemed competent in the literature ranged from 28% (10/36) to 63% (37/59) [2,14,15,16,17,18,25], and those deemed incompetent ranged from 28% (44/158) to 72% (26/36) [2,14,15,17,18,20,24]. Four studies had patients who fell into the mixed/other category [14,15,18,25]. Of those four studies, Clarke [15] had “no statements about capacity” for 18% (29/158) of the patients, Hussain [18] missed DMCA in 5% (3/59) of patients due to death, Astell [14] deemed some patients to have mixed competence, and Ranjith [25] deferred judgment in 15% (3/20) of the patients.

Use of a Capacity Interview only when necessary can prevent undue burden on patients and families. In our study, 79.3% of patients did not require a Capacity Interview, which is consistent with our earlier pilot study. If the DMCA is done well, the Capacity Interview is avoided in 80% of cases. A significant advantage to using the DMCA Model is the preservation of a patient’s autonomy and use of least intrusive and restrictive means to support them. This is echoed by the World Health Organization legislation, which recommends we should “recognize and protect the right to appropriate autonomy and self-determination” [26]. There are also likely economic benefits to avoiding declarations of incapacity and increasing lengths of stay while awaiting an increased level of care.

Some examples of problem-solving interventions to avoid Capacity Interview are utilizing informal and formal supports in personal decision making to mitigate the problems a patient may be experiencing. As mentioned in the methods, the patient leaving the stove on can be mitigateded with meals on wheels. The patient forgetting medication can be addresseded by adding homecare for medication assistance. The patient falling when getting out of the bath can be assisted by homecare for bathing assistance. The patient forgetting to pay bills can be helped by family oversight.

Incapacity based on advanced age, sex, or presence of dementia ought not be assumed [27,28]. Rather, care must be exercised to prevent undue Capacity Interviews without treating the underlying clinical conditions of patients admitted to the hospital for a short or long period of time [27,28]. This was evidenced in one record, in which an acutely ill patient with dementia and urinary tract infection (UTI) had an extended stay. An unwarranted Capacity Interview was performed without prior attempts at resolving the underlying condition and a wrongful declaration of incapacity was made. The patient regained some aspects of capacity after resolution of the underlying UTI. Avoidance of unnecessary Capacity Interviews is essential as it is a very intrusive process that can result in anxiety and stress to the patient. If the DMCA process had been used correctly, the patient would have been stabilized, and a Capacity Interview, which should always be a last resort after failure of problem-solving to address the issue, would have been avoided. This case presents a valuable learning opportunity for HCPs.

Training can support the use of a standardized DMCA process [29]. Data analyses exhibited utilization of the Capacity Assessment Process Worksheet in 63.2% of the referrals, suggesting that training supported fidelity to the DMCA Model [11]. The published literature also indicates that documentation was not always complete but was better for procedures than for routine care [3,4,19,30]. With adherence to the DMCA Model and consistent documentation, accurate results can be obtained regarding its effectiveness. Capacity Interviews were conducted in only 20.7% of referrals. However, following the DMCAs, 48.2% (41/85) of those assessed were deemed to lack capacity. Thus, it would appear declarations of incapacity were made without Capacity Interviews being documented. This may be explained by less complex cases, e.g., in severe dementia where the lack of capacity is more evident. However, it still speaks to poor documentation.

The results from this research add to the sparse literature on the actual outcomes of DMCAs. While some studies document the number of DMCAs conducted, disciplines involved, and whether those assessed were determined to be capable or not, there is a paucity of literature on the DMCA process and ways in which fidelity to it can favorably impact declarations of incapacity and reduce unnecessary burdens and hardship.

The DMCA Model is implemented with ongoing training throughout many levels of care and settings in Alberta, Canada. Additional details on the Model are published separately [11]. It was first used in acute care but is now used in home living teams, supportive living, and continuing care across the province. It is supported by champions, mentoring teams, and Steering Committees with management representation. Education occurs yearly in terms of online modules or a 4-h workshop for new staff and refreshers for experienced staff. Details of the education are published separately [12]. The DMCA Model has been in place since 2006 and is sustainable. Periodically, we will reassess outcomes and fidelity to the DMCA Model. This will be particularly important as care moves from paper to electronic charting.

This study has several strengths and limitations. Of benefit is the real-world nature of the study and size of the sample (88 patient charts dated 2006–2020). Review of records associated with patients treated at an acute care facility committed to training HCPs in a standardized DMCA Model and process enabled the team to examine the uptake and impact of DMCA Model and fidelity to it. The study, however, is limited to referrals to our geriatric service and does not capture all the DMCAs performed at the hospital. As capacity is not a discharge diagnosis, it is impossible to capture this assessment in all charts. The study is further limited by what is documented in the chart. There may be capacity issues where the trigger was determined to not be sufficient, and as a result, the Capacity Assessment Process Worksheet was not initiated. Further, the outcomes are from an acute care site and may not be transferable across different settings, though the DMCA Model has been used in practice across different settings.

## 5. Conclusions

The study suggests that the DMCA Model implemented at Grey Nuns Community Hospital has positive impacts on the assessment of the capacity of those whose decision-making capacity in multiple domains has come into question. DMCAs conducted with fidelity reduced the need for the Capacity Interview and declarations of incapacity while simultaneously respecting patient autonomy and supporting patients in their decisions in accordance with the legislation. The study highlights the need to use a DMCA process with fidelity to avoid unnecessary declarations of incapacity.

Furthermore, this DMCA Model could be included in the hospital quality management system for continuous improvement. Further research is warranted to determine the impact of use of the DMCA Model in other practice environments.

## Figures and Tables

**Table 1:** Characteristics of patients referred for DMCAs, 2006–2020.

	% (n/N)
Age	76 years old (SD: 10.5; Range: 49–98)
Sex	Females: 51.1% (45/88) Males: 48.9% (43/88)
Patient’s Living Arrangements	
Home	97.5% (77/79)
Supportive Living/Long Term Care	2.5% (2/79)
DMCA performed	72.6% (61/84)
Diagnosis of Dementia	
With Dementia	43.2% (38/88)
Unspecified Cognitive Impairment	13.6% (12/88)
No Dementia	42.2% (38/88)

Note: The denominators ranged from 58–88, depending on data availability for the corresponding variable.

**Table 2:** Characteristics of DMCA Process, 2006–2020.

Presence of Valid Trigger for DMCA	
Yes	93.0% (80/86)
No	7.0% (6/86)
Number of Domains Identified (all)	
1	13.2% (9/68)
2	29.4% (20/68)
3	25.0% (17/68)
4	30.9% (21/68)
5	1.5% (1/68)
Domains Identified for DMCA	
Accommodation	88.2% (60/68)
Healthcare	83.8% (57/68)
Finances	61.8% (42/68)
Legal Affairs	36.8% (25/68)
Others (Employment, Social, Educational)	7.4% (5/68)
Number of Disciplines Involved in the DMCA	
None	1.7% (1/58)
1	43.1% (25/58)
2	24.1% (14/58)
3	24.1% (14/58)
4	6.9% (4/58)
Types of Disciplines Involved in the DMCA	
Social Worker	81.0% (47/58)
Occupational Therapist	53.4% (31/58)
Physicians	22.4% (13/58)
LPN/RN/NP	19.0% (11/58)
Medical Student	10.3% (6/58)
Geriatrics/Psychiatry	3.4% (2/58)
Physical Therapy	1.7% (1/58)
Number of Referrals Made During the DMCA Process	
None	3.4% (3/88)
1	61.4% (54/88)
2	27.3% (24/88)
3	4.5% (4/88)
4	3.4% (3/88)
Types of Referrals	
Geriatrics	87.5% (77/88)
Social Worker	25.0% (22/88)
Psychiatry	15.9% (14/88)
Occupational Therapist	9.1% (8/88)
Others (DCA, Ethics, Family Medicine, Neuropsychiatry)	4.6% (4/88)
Capacity Assessment Process Worksheet Used	
Yes	63.2% (55/87)
No	36.8% (32/87)
Team Conference Held	
Yes	28.9% (24/83)
No	71.1% (59/83)
Capacity Interview Done	
Yes	20.7% (18/87)
No	79.3% (69/87)
Final Disposition	
Has Capacity	23.5% (20/85)
Lacks Capacity	48.2% (41/85)
Capacity Assessment Not Required	8.2% (7/85)
Unknown	20.0% (17/85)

Note: The denominators ranged from 58–88, depending on data availability for the corresponding variable.

## Data Availability

Requests for deidentified data may be directed to the corresponding author.
